# Supplementary material for: Resolving the full spectrum of human genome variation using Linked-Reads
Source: Genome Res. 2019 Apr;29(4):635–45. doi: 10.1101/gr.234443.118 (PMC6442396; doi:10.1101/gr.234443.118)
Supplement: Supplemental Material [file supp_gr.234443.118_Supplemental_longranger-2.2.zip › longranger-2.2.tar.bz2!/lr2.2-public/lib/stan/cmdstan/stan/doc/api/html/functions_func_n.html]

Stan: Class Members - Functions


|  |  |
| --- | --- |
|  | Stan  2.5.0  probability, sampling & optimization |


- Main Page
- Related Pages
- Namespaces
- Classes
- Files

- Class List
- Class Index
- Class Hierarchy
- Class Members

- All
- Functions
- Variables
- Typedefs
- Enumerator
- Related Functions

- a
- b
- c
- d
- e
- f
- g
- h
- i
- j
- k
- l
- m
- n
- o
- p
- q
- r
- s
- t
- u
- v
- w
- x
- z
- ~


All Classes Namespaces Files Functions Variables Typedefs Enumerations Enumerator Friends Macros Pages

### - n -

- name()
  : stan::gm::argument
  , stan::gm::type\_name< T >
  , stan::gm::type\_name< bool >
  , stan::gm::type\_name< double >
  , stan::gm::type\_name< int >
  , stan::gm::type\_name< std::string >
  , stan::gm::type\_name< unsigned int >
  , stan::gm::var\_decl
  , stan::io::dump\_reader
  , stan::mcmc::base\_mcmc
- name\_vis()
  : stan::gm::name\_vis
- names\_i()
  : stan::io::dump
  , stan::io::var\_context
  , stan::json::json\_data
- names\_r()
  : stan::io::dump
  , stan::io::var\_context
  , stan::json::json\_data
- newline()
  : stan::io::csv\_writer
- next()
  : stan::io::dump\_reader
- nonbasic\_validate()
  : stan::gm::validate\_var\_decl\_visgen
- note()
  : stan::optimization::BFGSMinimizer< FunctorType, QNUpdateType, Scalar, DimAtCompile >
- null()
  : stan::json::json\_data\_handler
  , stan::json::json\_handler
- num\_chains()
  : stan::mcmc::chains< RNG >
- num\_dims()
  : stan::gm::expr\_type
- num\_kept\_samples()
  : stan::mcmc::chains< RNG >
- num\_params()
  : stan::mcmc::chains< RNG >
- num\_params\_i()
  : stan::model::prob\_grad
- num\_params\_r()
  : stan::model::prob\_grad
- num\_promotions()
  : stan::gm::function\_signatures
- num\_samples()
  : stan::mcmc::chains< RNG >
  , stan::prob::welford\_covar\_estimator
  , stan::prob::welford\_var\_estimator
- number\_double()
  : stan::json::json\_data\_handler
  , stan::json::json\_handler
- number\_long()
  : stan::json::json\_data\_handler
  , stan::json::json\_handler
- number\_unsigned\_long()
  : stan::json::json\_data\_handler
  , stan::json::json\_handler


---

[ Stan Home Page ]

*© 2011–2014,
Stan Development Team.*
